# Yoga and breathing technique training in patients with heart failure and preserved ejection fraction: study protocol for a randomized clinical trial

**DOI:** 10.1186/s13063-018-2802-5

**Published:** 2018-07-28

**Authors:** Carla Pinheiro Lopes, Luiz Claudio Danzmann, Ruy Silveira Moraes, Paulo José Cardoso Vieira, Francisco França Meurer, Douglas Santos Soares, Gaspar Chiappa, Luciano Santos Pinto Guimarâes, Santiago Alonso Tobar Leitão, Jorge Pinto Ribeiro, Andreia Biolo

**Affiliations:** 1 School of Medicine, Post-Graduate Program in Health Sciences: Cardiology and Cardiovascular Sciences, Federal University of Rio Grande do Sul, Ramiro Barcelos, 2400, 2nd floor - Rio Branco, Porto Alegre, RS CEP 90035-903 Brazil; 2 Cardiovascular Division, Hospital de Clínicas de Porto Alegre, Porto Alegre, RS Brazil; 3 LaFIEx - Laboratory of Pathophysiology of Exercise, Hospital de Clínicas de Porto Alegre, Porto Alegre, RS Brazil; 4 School of Physical Education, Lutheran University of Brazil – ULBRA, Canoas, RS Brazil; 5 School of Medicine, Lutheran University of Brazil – ULBRA, Canoas, RS Brazil; 6 Unit of Biostatistics, Hospital de Clínicas de Porto Alegre, Porto Alegre, Brazil

**Keywords:** Yoga, Respiratory techniques, Heart failure with preserved ejection fraction, Randomized controlled trial, Autonomic system, Maximal inspiratory pressure, Maximal oxygen uptake

## Abstract

**Background:**

Current therapies for heart failure (HF) are followed by strategies to improve quality of life and exercise tolerance, besides reducing morbidity and mortality. Some HF patients present changes in the musculoskeletal system and inspiratory muscle weakness, which may be restored by inspiratory muscle training, thus increasing respiratory muscle strength and endurance, maximal oxygen uptake (VO_2_), functional capacity, respiratory responses to exercise, and quality of life. Yoga therapies have been shown to improve quality of life, inflammatory markers, and peak VO_2_ mostly in HF patients with a reduced ejection fraction. However, the effect of different yoga breathing techniques in patients showing HF with a preserved ejection fraction (HFpEF) remain to be assessed.

**Methods/design:**

A PROBE (prospective randomized open blinded end-point) parallel-group trial will be conducted at two specialized HF clinics. Adult patients previously diagnosed with HFpEF will be included. After signing informed consent and performing a pre-test intervention, patients will be randomized into three groups and provided with either (1) active yoga breathing techniques; (2) passive yoga breathing techniques (pranayama); or and (3) control (standard pharmacological treatment). Follow-up will last 8 weeks (16 sessions). The post-intervention tests will be performed at the end of the intervention period for analysis of outcomes. Interventions will occur continuously according to patients’ enrollment. The main outcome is respiratory muscular resistance. A total of 33 enrolled patients are expected. The present protocol followed the SPIRIT guidelines and fulfilled the SPIRIT checklist.

**Discussion:**

This trial is probably the first to assess the effects of a non-pharmacological intervention, namely yoga and specific breathing techniques, to improve cardiorespiratory function, autonomic system, and quality of life in patients with HFpEF.

**Trial registration:**

REBEC Identifier: RBR-64mbnx (August 19, 2012).

Clinical Trials Register: NCT03028168. Registered on 16 January 2017).

**Electronic supplementary material:**

The online version of this article (10.1186/s13063-018-2802-5) contains supplementary material, which is available to authorized users.

## Background

Several heart failure (HF) patients have limited physical activity due to early fatigue and dyspnea, which have been associated with low oxygen uptake and inspiratory muscle dysfunction, suggesting that physical deconditioning and respiratory muscle weakness might be underlying attenuated increased ventilation during hyperpnoea [1].

Over the last years, a reduced performance capacity has been associated with some additional factors that might be connected to the limited exercise response. Some studies have focused on the role of maximal inspiratory pressure (PI_max_) and inspiratory muscle endurance, which have been associated to low quality of life (QoL) and worse clinical prognosis [2–4]. In a randomized placebo-controlled trial with HF patients with reduced ejection fraction (HFrEF) and inspiratory muscle weakness (IMW), inspiratory muscle training markedly improved inspiratory muscle strength and endurance, incrementing submaximal and maximal functional capacity as well as QoL. In addition, heart rate variability (HRV) is also decreased by cardiovascular diseases and might result either from a direct central up-regulation of cardiovagal activity, or as a secondary effect of baroreceptor activation, or changes in respiration [5].

However, respiration has not been emphasized, despite evidence that breathing characteristics (rate and amplitude) markedly affect beat-to-beat cardiovascular variability [6–8]. Within this context, yoga breathing might combine abdominal, thoracic, and clavicular breathing phases in order to maximize breathing volume and therefore increase oxygen uptake. It has been reported that very and moderately slow yoga breathing creates a significant change in HRV frequency bands along and after exercise in healthy men and women [9, 10]. Recently, a study reported the comparative effectiveness of several forms of lifestyle modifications and found smoking cessation and yoga to be the most effective forms of cardiovascular disease prevention [11]. Furthermore, yoga techniques without breathing control have shown to improve oxygen uptake in patients showing HF, especially HFrEF [12]. However, even considering that almost half of HF patients show heart failure with preserved EF (HFpEF), few studies have been performed with such patients. It has been recently demonstrated that HFpEF induces significant molecular, mitochondrial, histological, and functional alterations in the diaphragm and soleus, which were attenuated by exercise training [13].

With respect to cardiovascular disease and aging, several authors have shown a significant reduction of HR variability in the frequency ranges associated with breathing by using spectral analysis of HR and respiration [14, 15]. Therefore, the present randomized clinical trial will be conducted primarily in order to test the hypothesis that an 8-week program of yoga and specific breathing techniques with various respiratory rhythms might be associated with improved inspiratory muscle responses. Secondly, changes in functional capacity, oxygen uptake efficiency slope, circulatory power, oscillatory ventilation, oxygen uptake kinetics in the recovery period, distinct features of the autonomic nervous system, natriuretic peptides, echocardiographic measurements, and QoL will be assessed in HFpEF patients, both with and without IMW.

## Methods/design

### Study design

A PROBE (prospective randomized open blinded end-point) parallel-group trial with three groups will be conducted at two specialized HF clinics (HF Clinic at Hospital de Clínicas de Porto Alegre (HCPA), RS, Brazil, and the HF ambulatory at Hospital ULBRA-Mãe de Deus, Canoas, RS, Brazil). The researchers will be divided according to their specific role in this study as (1) interveners – those performing the protocol interventions (blinded for outcomes, but not for groups); (2) medical appraiser – responsible for performing clinical tests (blinded for groups, but not for outcomes); and (3) analysts – those responsible for the statistical analyses (blinded for both groups and outcomes). The individuals will be instructed to avoid talking with the research team about protocol intervention and clinical trials.

### Inclusion and exclusion criteria

Adult patients aged from 45 to 75 years diagnosed with HFpEF, functional capacity class II and III, and who are being treated at a specialized HF clinic will be eligible. HF diagnosis will be established through medical history (signs and symptoms), echocardiographic findings (left ventricular ejection fraction ≥ 50%) [16], and medical records confirming management for HF.

Exclusion criteria are unstable angina, myocardial infarction, or cardiovascular surgery within the previous 3 months, active orthopedic or infectious disease, and treatment with steroids, hormones, or cancer chemotherapy. Additionally, pulmonary disease (forced vital capacity < 80% predicted and/or forced expiratory volume for 1 s < 70% predicted) [5, 17], significant mitral or aortic valve diseases, record of exercise-induced asthma, and active smoking will also be exclusion criteria. After selection, the discontinuous criteria are decompensated HFpEF, more than two consecutive absences in the intervention groups, and expressed willingness to discontinue at any time of the study.

### HF clinic and team approach

The two HF clinics participating in this trial are staffed by a multidisciplinary team of cardiologists, physical educators, physiotherapists, and nurses. On average, patients will start the protocols as soon as they conclude preclinical testing and will be randomized either to one of the intervention groups, or to the control group. In order to strengthen adherence to the study, the team will keep in touch by phone with the patients to reinforce their participation and confirm attendance at scheduled tests and interventions, or to understand the reasons for possible absences. Both centers have facilities that allow the performance of the yoga interventions described in the present study.

### Sample size

For sample size calculation, the higher the effect size and lower standard deviation in relation to the effect, the lower the sample size required to confirm the result. Thus, based on previous studies, the sample size was calculated using inspiratory muscle pressure as endpoint [18]. Considering a difference among treatments (effect size) of 15 cm H_2_O and a standard deviation (SD) of 12 cm H_2_O in the PI_max_, representing a 1.2 ratio (effect size/SD), using an α = 0.05 and power of 80%, nine patients would have to be included per group. In addition, considering a potential loss in patient follow-up of between 10% and 20%, the sample was set to 11 patients per group.

### Study protocol

Eligible patients will be initially evaluated by their medical history, prior HFpHF diagnosis, physical tests, resting electrocardiogram, two-dimensional echocardiogram, protocols of pulmonary function and inspiratory muscle function, cardiopulmonary exercise testing (CPET), 6-min walk test, Minnesota QoL, N-terminal pro b-type natriuretic peptide (NT-proBNP), and HRV frequencies (Holter 24 h). All of these evaluations will be detailed in the appropriate sections. These records and personal information will be securely stored in HCPA facility and accessed only by assistants and researchers.

**Fig. 1:**
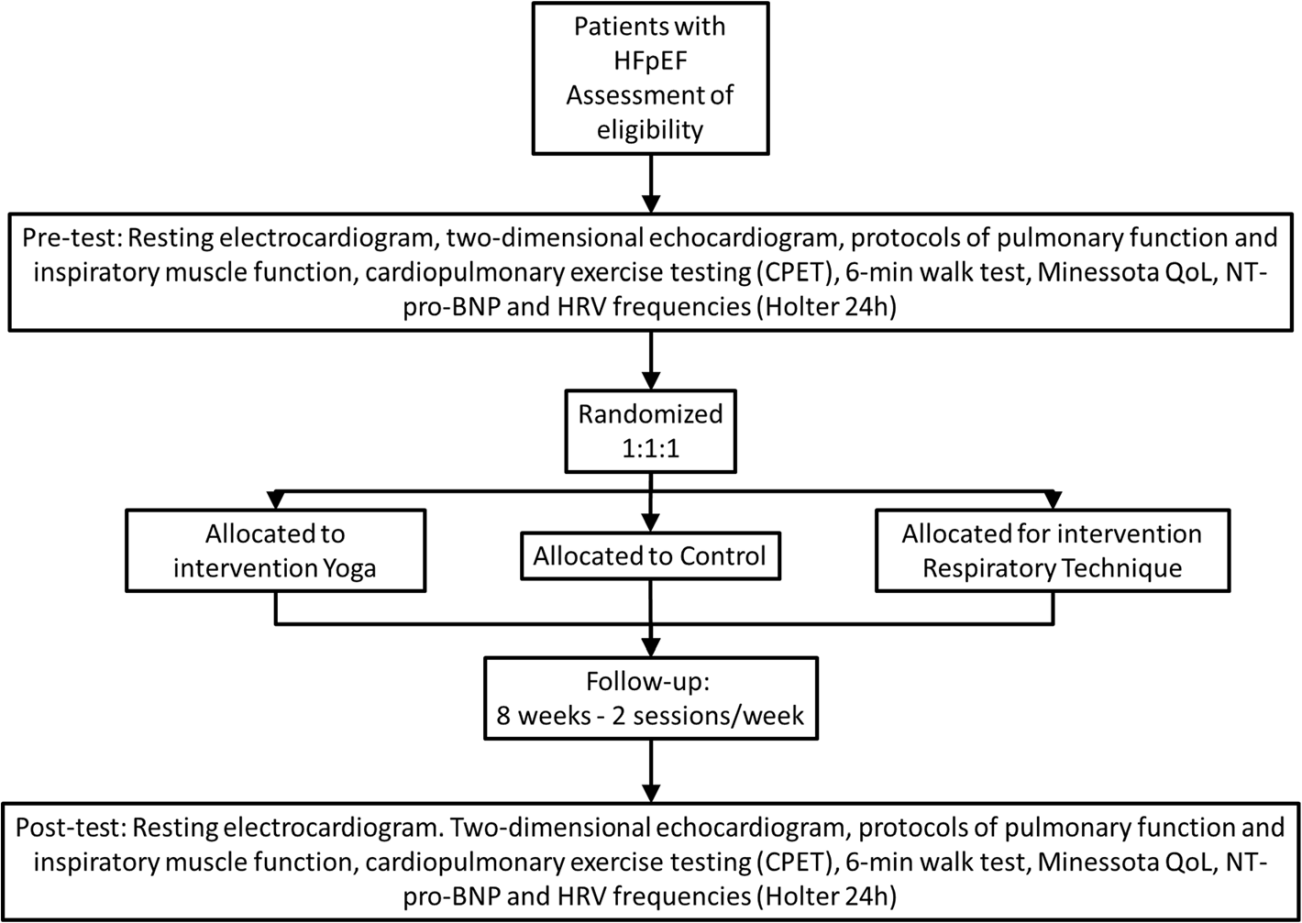
Flowchart of study participation and intervention

After signing informed consent and pre-test intervention forms, patients will be randomized into three groups as follows (Fig. 1). The present protocol followed the SPIRIT guidelines and fulfilled the SPIRIT checklist (Additional file 1):

#### Yoga – active breathing technique

Active protocol with yoga body movements (*àsanas*) performed along with respiratory technique without contentions, current and vigorous (*ujjayi*), observing a respiratory frequency of 15–20 respiratory cycles per minute. This session should last around 45 min.

#### Yoga – passive breathing technique (*pranàyama*)

Passive protocol, seated patient, no significant body movements. Yoga breathing technique with alternate nostril breathing (*viloma pranàyama*) uses diaphragmatic breathing, both current and combined with inspiratory and expiratory retentions, observing a slow respiratory frequency of between 5 and 8 respiratory cycles per minute. This session will last approximately 45 min.

A standardized 7-min final relaxation will be performed and will be common to both study intervention protocols.

#### Control group (standard pharmacological treatment)

Patients will be oriented to keep their pharmacological routine and daily activities with no structured exercises. They will have to return to the hospital for post-testing after 8 weeks of randomization. After final assessment, all patients, including those in the control group, will be invited to participate in the study breathing activities at the outpatient wards of this trial.

**Fig. 2:**
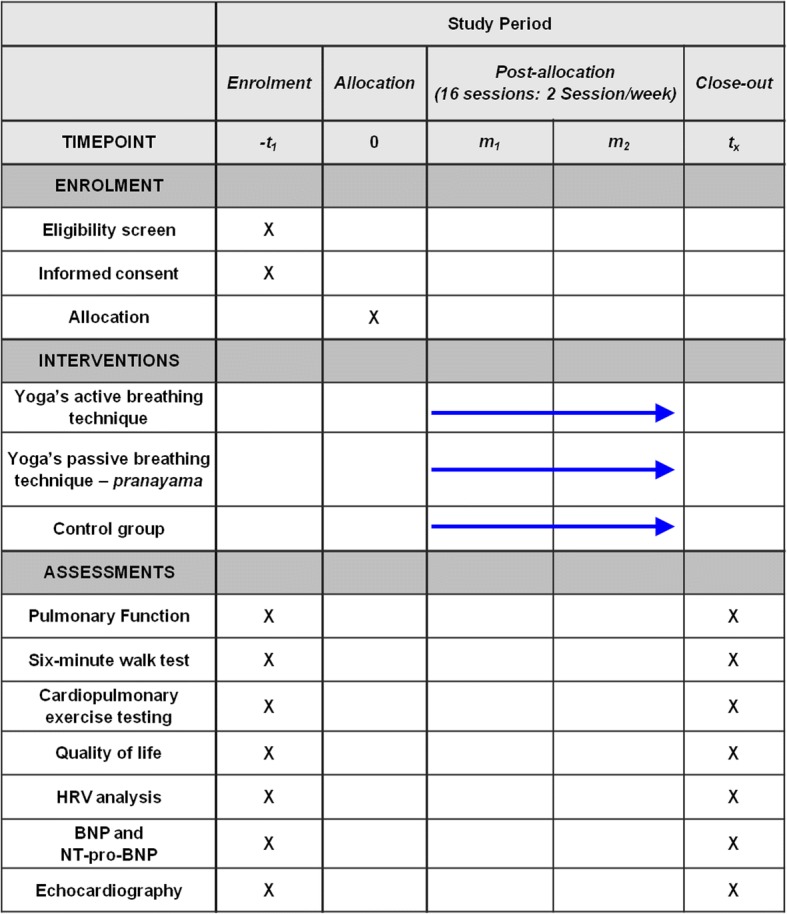
Schedule of enrollment, interventions, and assessments

Intervention will last 8 weeks (16 sessions). The post-intervention tests will be performed at the end of the intervention period for the evaluation of endpoints (Fig. 2). To ensure security, data will be stored both in the main researcher’s and study coordinator’s computers, as well as being added to a virtual drive. Researchers, study coordinators, doctors, and statisticians will be allowed data access. Interventions will occur at specific facilities in HCPA (physiatrist sector) and in ULBRA University hospital (medical school) according to patient enrollment. Protocol deviation and adverse events involving individual participants in clinical studies in the HCPA are recorded in the strategic adviser system available in the hospital intranet. Deviations and adverse events occurring at ULBRA will be recorded in the same system as the HCPA. The auditing procedures will occur randomly or designated by the ethical committee for research analysis.

The HFpEF diagnosis will be performed by trained cardiologist experts in the HF field according to the following criteria: HF patient showing an ejection fraction ≥ 50% and E/E’ index of > 8. In addition, other data will be considered for diagnosis, such as left atrial volume > 40 mL/m^2^, atrial fibrillation, and left ventricular mass index. The diagnosis will be confirmed by B-type natriuretic peptide (BNP) levels > 200 pg/mL or NT-proBNP > 220 pg/mL. Patients will be classified either as HFpEF with pseudonormal filling pattern (moderate, grade II) when 8 < E/E’ < 15; or as HFpEF with restrictive left ventricular filling pattern (severe, grade III) when E/E’ > 15. The HF functional classification will be assessed by the New York Heart Association (NYHA) classification system.

#### Pulmonary function

Inspiratory muscle function testing will be performed using a pressure transducer (MVD-500 V.1.1 Microhard System, Globalmed, Porto Alegre, RS, Brazil) connected to a system with two unidirectional valves (DHD Inspiratory Muscle Trainer, Chicago, Illinois, USA). Maximal static inspiratory pressure will be determined in deep inspiration from residual volume against an occluded airway, with a minor air leak (2 mm). The highest pressure within six measurements will be used for analysis. The PI_max_ measurement will be performed at rest and at the 5th and 10th min after CPET. Predicted values will be corrected for age, sex, and weight [14]. Additionally, in order to determine the inspiratory muscle endurance, an incremental test will be used in which patients will breathe continuously through a mouthpiece connected to a measure device. The patients will use an initial load of 50% PI_max_, and increments of 10% PI_max_ will be added every 3 min until the patient is unable to continue breathing. The highest inspiratory pressure that the individual is able to sustain for at least 1 min (Pth_max_) will be considered as the measure for inspiratory muscle endurance and will be expressed as a rate of maximal inspiratory pressure (Pth_max_/PI_max_). In the second stage of the protocol, individuals will breathe against a constant inspiratory submaximal load equivalent to 80% Pth_max_, and the time elapsed to task failure will be defined as the inspiratory endurance time. The Powerbreathe K5 will be used for patients with IMW and for those with normal respiratory fraction, for age and sex [15, 16].

#### Six-minute walk test

The maximum distance covered during the walk test will be used to assess submaximal functional capacity [19]. Patients will self-grade their degree of dyspnea during the test using the Borg scale [20].

#### Cardiopulmonary exercise testing

Maximal functional capacity will be evaluated using an incremental exercise test, with expired gas analysis, on a treadmill (INBRAMED10200, Porto Alegre, RS, Brazil), using a ramp protocol, starting at a speed of 2.4 km·h^− 1^ and 2% slope, with 20-s increments of speed (0.1 to 0.2 km·h^− 1^) and 60-s increments in slope (0.5% to 1.0%) to reach volitional fatigue at approximately 10 min. Twelve-lead electrocardiographic tracings will be obtained every minute (Nihon Khoden Corp., Tokyo, Japan). Blood pressure will be measured every 2 min with a standard cuff sphygmomanometer. Metabolic and ventilatory variables will be measured along and after exercise by 20-s mean aliquots, by a computer-aided gas analyzer (Total Metabolic Analysis System, TEEM 100, Aero Sport, Ann Arbor, Michigan, USA), previously validated [21]. Peak oxygen uptake (VO_2_ peak) will be considered as the highest VO_2_ value calculated in a 20-s period exercise. Maximal circulatory power will be calculated as the product of VO_2_ peak and peak systolic pressure [22]. Throughout incremental exercise, oxygen uptake will be plotted against the logarithm of total ventilation, and the oxygen uptake efficiency slope will be determined [23].

#### QoL

QoL will be assessed using the Minnesota Living with Heart Failure Questionnaire [24]. Overall scores and the separate effects of physical and psychological perceptions of QoL will be analyzed.

#### HRV analysis

Twenty-four-hour ECG recordings will be obtained with a SEER Light digital recorder (GE Medical Systems Information Technologies, Milwaukee, WI, USA). The recorded data will be analyzed using a MARS 8000 analyzer (Marquete Medical Systems, Milwaukee, WI, USA) [25, 26]. Briefly, time domain and frequency domain analyses of HRV will be performed according to the recommended by the European Society of Cardiology and North American Society of Pacing and Electrophysiology [27]. For time domain analysis, the following 24-h indices will be calculated: mean of all normal inter-beat (RR) intervals, standard deviation of all normal RR intervals, root mean square of successive differences of adjacent RR intervals, and rate of successive differences between normal adjacent RR intervals above 50 ms. For frequency domain analysis, the following spectral components will be calculated: low frequency (0.04–0.15 Hz), high frequency (0.15–0.5 Hz), and low-to-high frequency ratio. Spectral components will be expressed in absolute values (ms^2^ Hz) and in normalized units. Heart rate spectral analysis and time domain indices will be calculated using 5-min segments at rest during active breathing (*ujjayi*) and passive breathing (*pranàyama).*

#### BNP and NT-proBNP

Since this trial will be performed at two centers, both BNP or NT-proBNP methods will be used, yet the same biomarker (either BNP or NT-proBNP) will be employed for pre- and post-tests in any given patient. The BNP test will be performed on the clinical specimen using blood serum and a chemiluminescence analysis method (Advia Centaursiemens). The NT-proBNP test will be performed on a clinical specimen using blood serum and a sandwich-type electrochemiluminescence analysis method (Cobas E601-Roche).

#### Echocardiography

Usual and additional echocardiographic measures, such as volumetric gradients and diastolic parameters, will be analyzed in the selection of eligible patients (ejection fraction calculated by Teichholz method, ≥ 50%) [16] and after intervention by using two-dimensional echocardiograms (Philips IEE 33).

### Randomization

Patients who meet the eligibility criteria will be invited to participate in the study; those who accept to participate will sign a written informed consent form. Following the conclusion of pre-intervention tests, the researcher will inform the biostatistics center at HCPA, which is in charge of the randomization list, and participants will be allocated to either yoga (active) or yoga (passive) or control group in a 1:1:1 ratio using a pre-generated simple randomization list.

### Sociodemographic and clinical variables

A structured questionnaire will be filled by all study participants to collect sociodemographic data and clinical parameters (age, sex, education, current occupation or previous occupation when retired, daily routine, etiology and HF duration, presence of comorbidities such as hypertension, atrial fibrillation and aortic or mitral valve disease, history of smoking and alcohol intake, medications, hospitalizations, previous illnesses, presence of angina, stent placements, history of acute myocardial infarction, NYHA functional class, echocardiographic data). Weight, height, body mass index [28], vital signs, and electrocardiogram at rest will be evaluated in the first and last CPET.

Patients should be instructed to maintain their activities of daily living and eating routine, as established on the date of enrollment. Any change in pharmacological management should also be reported and recorded in the study.

### Endpoints

The primary endpoints are inspiratory muscle strength by measuring maximal inspiratory pressure (PI_max_).

Secondary endpoints include (1) vagal activity in resting and exercising (HRV); (2) peak VO_2_; (3) QoL Minnesota scores as a specific inventory for patients with HF; (4) functional capacity (NYHA Classification); (5) volumetric ratios of left atrial and diastolic pressure gradients on echocardiography; and (6) changes in BNP/NT-proBNP tests between pre- and post-intervention measurements.

### Independent/exposure variables and confounding

In both intervention groups (Active breathing technique or Passive breathing technique) the main independent variable includes respiratory management with different rhythms.

The effect of each intervention over the trial endpoints must be controlled for the confounders of concomitant physical exercise or co-intervention, and any changes regarding medications or non-pharmacological treatment.

### Statistical analysis

Initially, a descriptive analysis will be performed and data will be expressed as absolute and relative frequency, besides mean and standard deviation or quartiles, accordingly. The treatment groups will be compared using the generalized estimating equation, specific for repeated measurements, in order to compare the effects (means) across the three groups and the two times, in addition to the group × time interaction. The generalized estimating equation matrix of robust estimator covariance and exchangeable work correlation matrix will be used if normal distribution is found and will be analyzed by an identity binding function. In contrast, if an asymmetrical distribution is found, data will be analyzed using a gamma distribution linked to a logarithmic function. When significant, the factors under study will be compared by Bonferroni’s post-hoc test. Correlations will be described by the Pearson’s or Spearman’s test. The PASW18 (version 18.0, SPSS, Chicago, Illinois, USA) will be used in this analysis.

## Discussion

Patients showing HFpEF have poor functional capacity despite preserved systolic function, and little has been added to the treatment of those patients. Recent research indicates that patients with restrictive ventilation, poor functional capacity, or peripheral abnormalities have more symptoms of exercise intolerance. Therapeutic strategies are necessary to improve exercise tolerance by targeting the integrated functions of these systems [29].

Traditionally, yoga is a complex physical practice associated with specific respiratory techniques [30]. Currently, it has been recommended in risk reduction programs [11] and cardiovascular rehabilitation [31], and its benefits include well-being, cognitive and motor improvement, positive effects in the treatment of hypertension [30], decreasing inflammatory process [32], and improved functional capacity of HFrEF patients [12]. In HFpEF patients, there are scarce reports based on the functional and neuromuscular properties during an acute or adaptive response to physical yoga and/or breathing practices.

This trial will assess the effects of yoga and specific breathing techniques as a non-pharmacological intervention in order to improve patients with HFpEF by promoting a decrease in the dyspnea degree during rest and exercise intolerance, which may represent a new treatment possibility for those patients. To this end, the NYHA classification will be assessed.

In order to reduce trial bias, this study has been carefully designed according to the PROBE concept [33], in which patients will be randomly allocated into intervention groups in a researcher-independent way, since the statistics department in the HCPA facility has a central phone for randomization. Furthermore, the performance bias will be controlled by single blinding according to researcher participation on trial.

Finally, all the results obtained in this trial will be published fulfilling all criteria of the CONSORT 2010 statement and its extension to non-pharmacological intervention.

### Trial status

This trial is currently in the patient selection and intervention stages. To date, 20 patients have been enrolled, 18 of whom have completed the trial.

## Additional file

Additional file 1: SPIRIT Table. SPIRIT checklist of the protocol study. (PDF 182 kb)

## Abbreviations

BNP: B-type natriuretic peptide
CPET: cardiopulmonary exercise testing
HCPA: Hospital de Clínicas de Porto Alegre
HFpEF: heart failure with preserved ejection function
HFrEF: heart failure with reduced ejection fraction
HRV: heart rate variability
IMW: inspiratory muscle weakness
NT-proBNP: N-terminal pro b-type natriuretic peptide
NYHA: New York Heart Association
PI_max_: maximal static inspiratory pressure
Pth_max_: maximal inspiratory pressure kept for 1 min throughout the incremental test
QoL: quality of life
RR: normal inter-beat
VO_2_: oxygen uptake
