# Supplementary material for: Yoga and breathing technique training in patients with heart failure and preserved ejection fraction: study protocol for a randomized clinical trial
Source: Trials. 2018 Jul 28;19:405. doi: 10.1186/s13063-018-2802-5 (PMC6064087; doi:10.1186/s13063-018-2802-5)
Supplement: Supplementary file 1 — SPIRIT Table. SPIRIT checklist of the protocol study. (PDF 182 kb) [file 13063_2018_2802_MOESM1_ESM.pdf]

SPIRIT

STANDARD PROTOCOL ITEMS: RECOMMENDATIONS FOR INTERVENTIONAL TRIALS

SPIRIT 2013 Checklist: Recommended items to address in a clinical trial protocol and related documents*

Section/item	Item No	Description	Addressed on page number
**Administrative information**			
Title	1	YOGA AND BREATHING TECHNIQUES TRAINING IN PATIENTS WITH HEART FAILURE AND PRESERVED EJECTION FRACTION: Study Protocol for a randomized clinical trial	1
Trial registration	2a	REBEC - RBR-64mbnx;<br>CLINICAL TRIAL - NCT03028168	10
Protocol version	3	REBEC - (19 August 2012) and the last update was made on 02 September 2013<br>CLINICAL TRIALS - (16 January 2017)	10
Funding	4	This study is supported by the FIPE/HCPA (Research and Education Funds from the Hospital de Clínicas de Porto Alegre). The grant number was recorded as 11-0069.	11

Roles and responsibilities	5a	Carla Pinheiro Lopes – Hospital de Clínicas de Porto Alegre – Study Coordinator<br>Luiz Claudio Danzmann – Hospital ULBRA/Mãe de Deus - co-Investigator<br>Ruy Silveira Moraes – Hospital de Clínicas de Porto Alegre - co-investigator<br>Paulo José Cardoso Vieira – Hospital de Clínicas de Porto Alegre – study assistant<br>Francisco França Meurer – Hospital de Clínicas de Porto Alegre - study assistant<br>Douglas Santos Soares – Hospital de Clínicas de Porto Alegre - study assistant<br>Gaspar Chiappa – Hospital de Clínicas de Porto Alegre - study assistant<br>Luciano Santos Pinto Guimarâes – Hospital de Clínicas de Porto Alegre - statitician<br>Santiago Alonso Tobar Leitão - Hospital de Clínicas de Porto Alegre - co-investigator<br>Jorge Pinto Ribeiro – Hospital de Clínicas de Porto Alegre - Principal investigator (in memorian)<br>Andreia Biolo – Hospital de Clínicas de Porto Alegre - Principal investigator (current)	1
**Introduction**			
Background and rationale	6a	Current therapies for heart failure (HF) bring together strategies to improve quality of life and exercise tolerance, as well as to reduce morbidity and mortality. Some HF patients present changes in the musculoskeletal system and inspiratory muscle weakness, which may be restored by inspiratory muscle training, thus increasing respiratory muscle strength and endurance, maximum oxygen consumption (VO2), functional capacity, respiratory responses to exercise, and quality of life. Yoga therapies have been shown to improve quality of life, inflammatory markers, and VO2 peak in HF patients, mostly with reduced ejection fraction. However, the effect of different yoga breathing techniques in patients with HF with preserved ejection fraction (HFpEF) has yet to be assessed.	4
Objectives	7	Will be conducted in order to test the hypothesis that an 8-week program of Yoga and specific breathing techniques with different ventilatory rhythms could be associated with improvement in inspiratory muscle responses, functional capacity, oxygen uptake efficiency slope (OUES), circulatory power, oscillatory ventilation, kinetics of oxygen consumption in the recovery period, distinct features of the autonomic nervous system, natriuretic peptides, echocardiographic measurements, and quality of life in patients with HFpEF, with and without IMW.	4

Trial design	8	A PROBE (prospective randomized open blinded endpoint) parallel-group, trial with three groups will be conducted at two specialized HF clinics (HF Clinic at Hospital de Clínicas de Porto Alegre, and the HF Ambulatory at Hospital ULBRA-Mãe de Deus, Canoas, RS).	5
**Methods: Participants, interventions, and outcomes**			
Study setting	9	Hospital de Clínicas de Porto Alegre – Porto Alegre/RS – Brazil<br>Hospital ULBRA-Mãe de Deus – Canoas/RS – Brazil	5
Eligibility criteria	10	Adult patients aged from 45 to 75 years diagnosed with HFpEF, functional capacity class II and III, who are being treated at a specialized HF clinic will be eligible. HF diagnosis will be established by medical history (signs and symptoms), echocardiographic findings (left ventricular ejection fraction ≥ 50%) [16] and medical records confirming management for HF.<br>Exclusion criteria are unstable angina, myocardial infarction, or cardiovascular surgery within the previous three months; active orthopedic or infectious disease; and treatment with steroids, hormones, or cancer chemotherapy. Additionally, pulmonary disease (forced vital capacity <80% predicted and/or forced expiratory volume for 1 s <70% predicted) [5, 17], significant mitral or aortic valve diseases, record of exercise-induced asthma, and active smoking. After selection, the discontinuous criteria are decompensated HFpEF, more than two consecutive missing in the intervention groups and expressed willing to discontinue at any moment of the study.	5

Interventions	11a	**Yoga – active breathing technique**. Active protocol with yoga body movements (*àsanas*) performed along with respiratory technique without contentions, current and vigorous(*ujjayi*), observing respiratory frequency (RF) of 15- 20 respiratory cycles per minute (rcpm). Session should last around 45 minutes.<br><br>**Yoga's passive breathing technique – *pranayama*.** Passive protocol, seated patient, no significant body movements. Yoga breathing technique, with alternate nostril breathing (*viloma pranàyama*), uses diaphragmatic breathing, both current and combined to inspiratory and expiratory retentions, observing slow RF, between 5-8 rcpm. Session will last approximately 45 minutes. A standardized 7-minute final relaxationwill be performed and will be commonto both study intervention protocols.<br><br>**Control group** (standard pharmacological treatment). Patients will be oriented to keep their pharmacological routine and daily activities, with no structured exercises. They will have to return to the hospital for post-testing after 18 weeks from randomization. After final assessment, all patients, including control group, will be invited to participate in the study breathing activities at the outpatient wards of this trial.<br><br>Intervention will take 8 weeks (16 sessions). The post-intervention tests will be performed at the end of the intervention period for evaluation of endpoints. Interventions will occur at specific facilities in HCPA (Physiatrist sector) and in ULBRA University Hospital (Clinical School) according to patients' enrollment.	5-6
	11b	Discontinuous criteria are: (a) HFpEF decompensated that justify the exclusion of study; (b) more than two consecutive missing in the intervention groups and; (c) if subject manifest intention to discontinue at any moment of the study.	5
	11c	To improve adherence to the study, the team maintains telephone contact to reinforce the subject's participation and confirm attendance at scheduled tests, interventions, or to understand the reasons for possible patients missing.	5
	11d	During the study period (8 weeks) is requested for subjects not to change their medication, diet and physical activity patterns. In case of changes investigators must be informed.	8

Outcomes	12	The primary endpoints are inspiratory muscle strength by measuring maximal inspiratory pressure (PImax). Secondary endpoints include: (a) Vagal activity in resting and exercising heart rate variability- HRV); (b) peak VO2 (c) Quality of life Minnesota scores as a specific inventory for patients with HF; (d) Functional capacity **(NYHA** Classification); (e) Volumetric ratios of LA and diastolic pressure gradients on echocardiography; (f) Changes in BNP/NT-proBNP tests between pre- and post-interventions.	8
Participant timeline	13	Eligible patients will be initially evaluated by medical history, physical examination, resting electrocardiogram, two-dimensional echocardiogram, protocols of pulmonary function and inspiratory muscle function, cardiopulmonary exercise testing (CPET), 6-min walk test, Minessota QoL, NT- pro-BNP e HRV frequencies (Holter 24h). All of these evaluations will be explained in details in the appropriate sections.<br>After signing informed consent and pre-test intervention, patients will be randomized into 3 groups.	5-8
Sample size	14	For sample size calculation, the higher the effect size and lower standard deviation in relation to the effect, the lower the sample size required to confirm the result. Thus, based on previous studies, sample size was calculated using inspiratory muscle pressure as endpoint [18]. Therefore, considering a difference among treatments (effect size) of 15 cm H2O and a standard deviation (SD) of 12 cm H2O in the PImax, representing a 1.2 ratio (effect size/SD), using an $\alpha = 0.05$ and power of 80%, nine patients would have to be included per group. In addition, considering a potential loss in patient's follow-up between 10-20%, the sample was set at 11 patients per group.	5
Recruitment	15	Screening of eligible subjects is performed by medical records on Hospital's database and by trained cardiologists who diagnose HFpEF on patients attending the ambulatory service from Hospital Universitário ULBRA-Mãe de Deus or Hospital de Clínicas de Porto Alegre	5

Methods: Assignment of interventions (for controlled trials)

Allocation:

Sequence generation	16a	Randomization was performed using function =RAND() on Excel software (Microsoft Office 2010) controlling the final group size to keep groups balanced.	8

Allocation concealment mechanism	16b	Implementation of the allocation sequence is performed after baseline pre-intervention exams by Call Center service from the Post-Graduation and Research Group at Hospital de Clínicas de Porto Alegre (GPPG/HCPA).	8
Implementation	16c	Statistician from GPPG/HCPA will answer the phone and allocate the subject into groups as follow: “Y” for Yoga group; “R” for Respiratory Technique Group and “C” for control group.	8
Blinding (masking)	17a	The researchers will be divided according to their specific role in this study: (i) interveners – who performed the protocols interventions – blinded for outcomes, but not for groups; (ii) medical appraiser – personal responsible for performing clinical tests – blinded for groups, but not for outcomes; and (iii) analyst – who is responsible for the statistical analyses – blinded both for groups and outcomes. The individuals will be instructed to avoid talking to the research team about protocol intervention and clinical trials.	5

Methods: Data collection, management, and analysis

Data collection methods	18a	After signing the informed consent, subjects diagnosed with HFpEF will be subjected to baseline exams (pre-randomization), as follow: NT-pro-BNP (≥ 300 ng/L), Minessota query (QoL), powerbreath, manovacuometer (PI max and PE max), ergospirometry (VO_2 max or pic), dopler echocardiogram (e/e’), and holter (HRV). After exams, subjects will be randomized and allocated into interventions groups or control group and followed up for 8 weeks. At the end of protocol subjects will be submitted to the same routine of exams. All data will be added to database which will be filled by trained assistant and checked by study investigators.	5-8
	18b	During protocol follow up, the importance of subject participation will be reinforced at every phone contact.	5
Data management	19	All data will be stored in principal investigator and study coordinator computer and added to virtual drive.	6; 11

Statistical methods	20a	Initially, a descriptive analysis will be performed and data will be expressed as absolute and relative frequency, besides mean and standard deviation or quartiles accordingly. The treatment groups will be compared using the Generalized Estimating Equations (GEE-GZLM), specific for repeated measurements, in order to compare the effects (means) across the three groups and the two times, in addition to the group x time interaction. The GEE Matrix of robust estimator covariance and exchangeable work correlation matrix will be used whether normal distribution is found and will be analyzed by an identity binding function. In contrast, whether an asymmetrical distribution is found, data will be analyzed using a gamma distribution linked to a logarithmic function. When significant, the factors under study will be compared by Bonferroni's post-hoc test. Correlations will be described by the Pearson’s or Spearman’s test. The PASW18 (version 18.0, SPSS, Chicago, Illinois, USA) will be used in this analysis.	9
	20b	Data will be adjusted for subgroup parameters when appropriated.	9
	20c	The treatment groups will be compared using the Generalized Estimating Equations (GEE-GZLM), specific for repeated measurements, in order to compare the effects (means) across the three groups and the two times, in addition to the group x time interaction. The GEE Matrix of robust estimator covariance and exchangeable work correlation matrix will be used whether normal distribution is found and will be analyzed by an identity binding function. In contrast, whether an asymmetrical distribution is found, data will be analyzed using a gamma distribution linked to a logarithmic function.	9
**Methods: Monitoring**			
Data monitoring	21a	Data monitoring will be performed by study coordinator and by principal investigator.	6
Harms	22	Registration of protocol deviation and adverse events involving subject’s participants in clinical studies in the HCPA is recorded in the system Strategic Adviser (SA), available in Hospital intranet. Deviations and adverse events occurring at HU-Ulbra-Mãe de Deus will be recorded in the same system from HCPA.	11
Auditing	23	Auditing procedures are random or by indication of the Ethical Committee for research analysis.	11
**Ethics and dissemination**			
Research ethics approval	24	This protocol was registered in the Brazilian Records of Clinical Trials (REBEC) with the identifier number: RBR-64mbnx (19 August 2012) and the last update was made on 02 September 2013; This protocol was also registered in ClinicalTrial.org with identifier number: NCT03028168	10

Protocol amendments	25	Every protocol modification will be communicated to ethical committee as descriptive amendment highlighting protocol alterations.	11
Consent or assent	26a	All collaborators were trained to introduce, show and take questions about the informed consent, exams to be taken and eventually discomforts associated to interventions before the signature of the informed consent.	11
Confidentiality	27	Subject's personal information will be kept securely stored in HCPA facility with access only to assistants and investigators.	11
Declaration of interests	28	No conflict of interest	10
Access to data	29	Investigators, Study coordinators, physicians and statistician.	11
Ancillary and post-trial care	30	All patients will be followed up after the end of protocol in the ambulatory services from both Hospital de Clínicas de Porto Alegre and Hospital Universitário ULBRA-Mãe de Deus.	11

*It is strongly recommended that this checklist be read in conjunction with the SPIRIT 2013 Explanation & Elaboration for important clarification on the items. Amendments to the protocol should be tracked and dated. The SPIRIT checklist is copyrighted by the SPIRIT Group under the Creative Commons "Attribution-NonCommercial-NoDerivs 3.0 Unported" license.
